# Efficacy of Combined Topical Timolol and Oral Propranolol for Treating Infantile Hemangioma: A Meta-Analysis of Randomized Controlled Trials

**DOI:** 10.3389/fphar.2020.554847

**Published:** 2020-10-08

**Authors:** Junbo Qiao, Junjie Lin, Dexin Zhang, Junhua Li, Changkuan Chen, Hongye Yu, Xiaodi Li, Bin Fang

**Affiliations:** Department of Hemangioma Surgery, The Third Affiliated Hospital of Zhengzhou University, Zhengzhou, China

**Keywords:** timolol, propranolol, infantile hemangioma, effective rate, meta-analysis

## Abstract

**Background:**

Oral propranolol has become the first-line treatment for infantile hemangioma (IH). However, combined therapy with topical timolol and oral propranolol has been proposed as a more effective IH treatment strategy. We aimed to compare the safety and efficacy of topical timolol, oral propranolol, and their combination for treating IH in a meta-analysis.

**Methods:**

Relevant randomized controlled trials (RCTs) were obtained after searching the PubMed, Embase, Cochrane’s Library, China National Knowledge Infrastructure, and WanFang databases. A random-effect model was used to pool the results.

**Results:**

Eight RCTs with 759 patients with IH were included in this meta-analysis. Treatment with topical timolol alone showed a similar response rate compared to oral propranolol (risk ratio [RR] = 0.97, p = 0.63), but resulted in fewer adverse events (RR = 0.36, p = 0.002). Combined treatment with topical timolol and oral propranolol showed a favorable response rate compared to treatment with oral propranolol (RR = 1.14, p = 0.03) or topical timolol (RR = 1.36, p = 0.01) alone. Moreover, combined treatment showed similar risks of adverse events compared to oral propranolol (RR = 0.80, p = 0.24) or topical timolol (RR = 1.31, p = 0.25) alone.

**Conclusions:**

Combined treatment with topical timolol and oral propranolol may be more effective than either single treatment strategy in patients with IH. Topical timolol alone conferred similar efficacy for IH compared to oral propranolol, but with less incidence of adverse events.

## Introduction

Infantile hemangioma (IH) is a common congenital vascular malformation that affects 4–5% of full-term infants (Itinteang et al., 2014; Grzesik and Wu, 2017). The incidence of IH is higher in females than in males, with an estimated ratio of 3–5:1 (Harter and Mancini, 2019; Wildgruber et al., 2019). Because of a natural history of spontaneous involution, most IHs do not need treatment (Harter and Mancini, 2019). However, some severe IH cases are associated with complications including bleeding, ulceration, or disfigurement, which require early interventions (Singh et al., 2019). Previous treatments for IHs involve multiple modalities, with glucocorticoids considered the medication of choice (Chinnadurai et al., 2016). Since the first-report of successful treatment of IH with oral propranolol in 2008, this non-selective β-blocker has become the mainstay for managing complex IHs (Leaute-Labreze et al., 2015; Yang et al., 2019). Although oral propranolol is effective and safe in most IH cases, it can cause severe adverse events, such as hypotension, bradycardia, hypoglycemia, sleep disturbance, and possibly central nervous system symptoms (Leaute-Labreze et al., 2016; Raphael et al., 2016; Thai et al., 2019). Timolol, another non-selective β-blocker, which can be applied topically in gel or in solution, has emerged as a novel IH treatment (Zheng and Li, 2018). Topical timolol may confer similar efficacy as propranolol for treating IHs, particularly the superficial type, but causes less adverse events (Cheirif-Wolosky et al., 2019). However, use of topical timolol for IH treatment is mainly based on clinical experience and evidence from observational studies, rather than randomized controlled trials (RCTs) (Wu et al., 2018). Combined therapy with topical timolol and oral propranolol has been proposed to be more effective for treating IHs compared to application of either component alone (Ge et al., 2016; Tong et al., 2016). However, clinical evidence to support this proposal remains limited. Therefore, in this study, we conducted a meta-analysis of head-to-head RCTs to systematically evaluate the efficacy and safety of topical timolol, oral propranolol, and their combination for treating IH.

## Methods

This meta-analysis was conducted according to the PRISMA (Preferred Reporting Items for Systematic Reviews and Meta-Analyses) statement (Moher et al., 2009) and the Cochrane Handbook for Systematic Review and Meta-analysis (Higgins and Green, 2011).

### Search Strategy

The PubMed, Embase, Cochrane’s Library (Cochrane Center Register of Controlled Trials), China National Knowledge Infrastructure (CNKI, http://www.cnki.net/), and WanFang (http://www.wanfangdata.com.cn/) electronic databases were searched for studies comparing the efficacy and safety among topical timolol, oral propranolol, and their combination for treating IH, using the terms “timolol” AND “propranolol” AND (“hemangioma” OR “haemangioma” OR “vascular malformation” OR “hemangiom*” OR “angiom*” OR “malformation*”). The search was limited to human studies with no restriction of publication languages. We also manually analyzed reference lists of the original and review articles. The final search was performed on June 3, 2020.

### Study Selection

Studies were included if they met the following criteria: (1) published as full-length articles with no restriction of languages; (2) reported as RCTs with a parallel design; (3) included patients with IH; (4) directly compared the efficacy and safety of at least two of the following treatment strategies, including topical timolol, oral propranolol, and their combination; (5) reported a treatment duration of at least one month; and (6) reported efficacy outcomes as response rates and safety outcomes of any adverse events that were confirmed to be related to the medications. The response rate was defined as a reduction in tumor size of at least 50% after treatment in accordance with the criteria proposed by Achauer et al., (1997). Reviews, preclinical studies, non-RCTs, and studies that did not report related outcomes were excluded from the current analysis.

### Data Extraction and Quality Assessment

Two authors independently performed literature searches, data extraction, and quality assessment according to the inclusion criteria. Discrepancies were resolved by consensus. Extracted data included the country where the study was performed, study design characteristics (blind or open-label), characteristics of the infantile patients (number of participants, sex, and age), lesion characteristics (distribution and clinical classification), and intervention characteristics (medications, routes, dosages, and treatment durations). We used the seven domains of the Cochrane risk of bias tool to evaluate the quality of the included studies (Higgins and Green, 2011), which included criteria concerning sequence generation, allocation concealment, blinding of participants and personnel, blinding of outcome assessors, incomplete outcome data, selective outcome reporting, and other potential threats to validity.

### Statistical Analysis

Dichotomous data were analyzed using risk ratios (RRs) with 95% confidence intervals (CIs). The Cochrane’s Q test was applied to evaluate the heterogeneity among the included studies, and significant heterogeneity was considered at p < 0.10 (Higgins et al., 2003). The I^2^ statistic, which describes the percentage of total variation across studies due to heterogeneity rather than chance, was also calculated to determine study heterogeneity (Higgins and Thompson, 2002). An I^2^ > 50% indicated significant heterogeneity among the trials. Pooled analyses were calculated using a random-effect model because this model incorporated the potential heterogeneity among the included RCTs and therefore could retrieve a more generalized result (Higgins and Green, 2011). Potential publication bias was assessed by visual inspection of the symmetry of the funnel plots and Egger’s regression asymmetry test (Egger et al., 1997). P values were two-tailed and statistical significance was set at 0.05. We used RevMan software for the meta-analysis and statistical study (Version 5.1; Cochrane, Oxford, UK) and Stata software (Version 12.0; Stata, College Station, TX).

## Results

### Search Results

**Figure 1:**
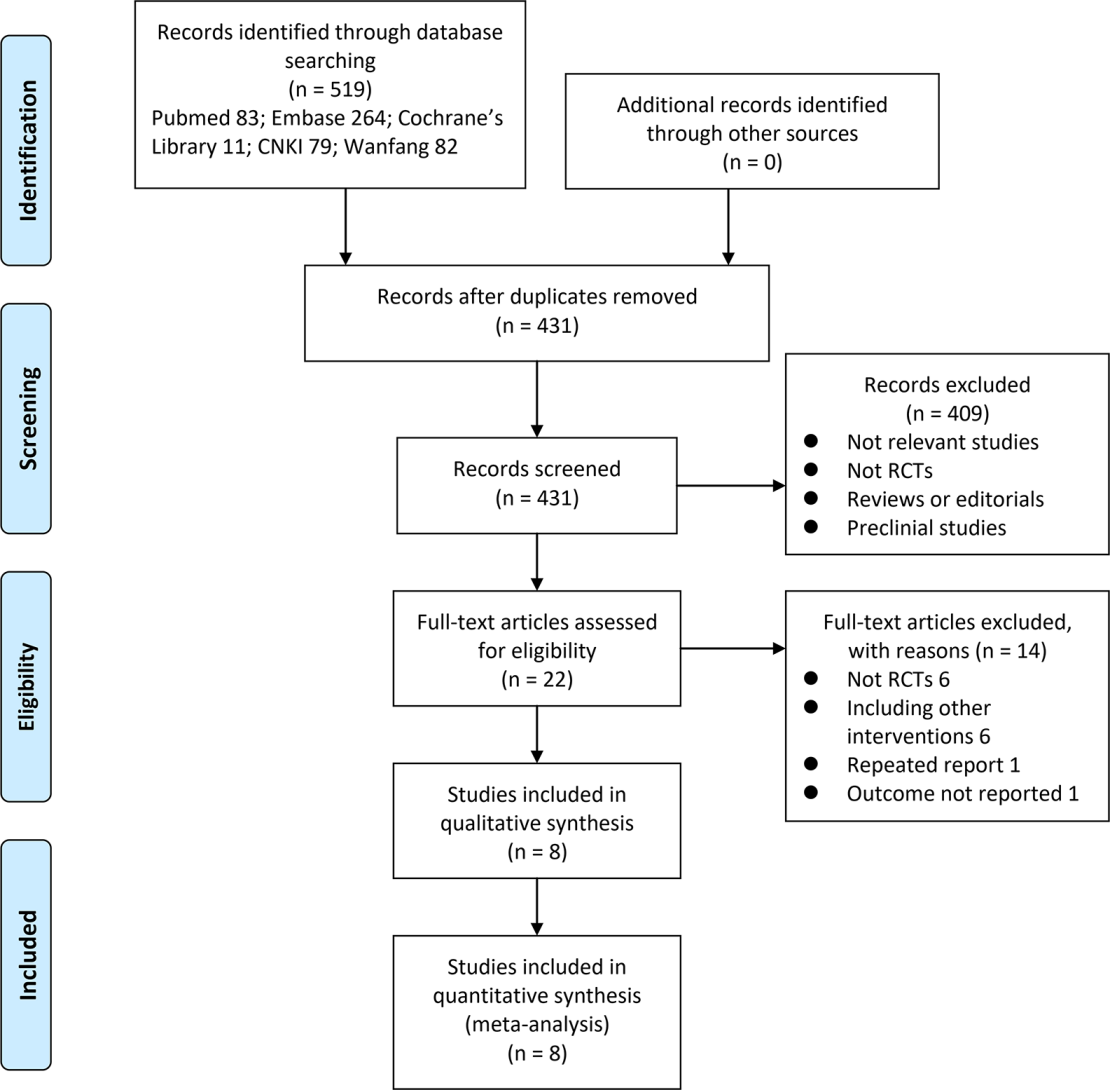
Flowchart of database search and study identification.

A total of 519 articles were identified through initial database searches, and 431 were obtained after excluding of duplications. Among them, 409 were excluded because they were not relevant studies based on title and abstract screening. Of the 22 potentially relevant articles, 14 articles were excluded because six were not RCTs, six did not compare the interested interventions, one was a repeated report of an already included RCT, and one did not report response rate for the treatment. Finally, eight RCTs were included (Zhao et al., 2013; Li et al., 2014; Chen et al., 2015; Gong et al., 2015; Li et al., 2016; Zhang et al., 2017; Du et al., 2018; Marey et al., 2018) (**Figure 1**).

### Study Characteristics

Overall, this meta-analysis included 759 patients with IH among eight RCTs (Zhao et al., 2013; Li et al., 2014; Chen et al., 2015; Gong et al., 2015; Li et al., 2016; Zhang et al., 2017; Du et al., 2018; Marey et al., 2018). The characteristics of the included RCTs are summarized in **Table 1**. Three RCTs were published in English (Gong et al., 2015; Li et al., 2016; Marey et al., 2018), and the other five were published in Chinese (Zhao et al., 2013; Li et al., 2014; Chen et al., 2015; Zhang et al., 2017; Du et al., 2018). All of the included studies were performed in China except one study which was performed in Egypt (Marey et al., 2018). The ages of the included patients varied from 1 to 18 months, with the percentage of males varying from 28–52%. With regards to the distributions of IH lesions, one study included patients with periocular hemangiomas (Marey et al., 2018), three included patients with IH in the facial area (Li et al., 2014; Gong et al., 2015; Li et al., 2016), and the remaining four studies included lesions in the face, scalp, neck, torso (20), scalp, perineum, and extremities (Zhao et al., 2013; Chen et al., 2015; Zhang et al., 2017; Du et al., 2018). The IHs were superficial in five studies (Zhao et al., 2013; Li et al., 2014; Gong et al., 2015; Zhang et al., 2017; Marey et al., 2018) and of mixed clinical classifications in the other threes studies (Chen et al., 2015; Li et al., 2016; Du et al., 2018). Three studies compared the efficacies between topical timolol and oral propranolol (Zhao et al., 2013; Li et al., 2014; Zhang et al., 2017), three compared the efficacies between oral propranolol and a combined therapy with topical timolol and oral propranolol (Chen et al., 2015; Li et al., 2016; Marey et al., 2018), the other two studies compared the efficacies among the three treatments (Gong et al., 2015; Du et al., 2018). The follow-up durations varied from one to eight months.

**Table 1:** Characteristics of the included RCTs.

Study	Country	Design	Sample size	Age	Male	Sites	Clinical classification	Intervention and dosage	Treatment duration
				months	%				months
Zhao et al., 2013	China	R	141	2–13, median 4.9	28.4	Face and neck (94), torso (20), scalp (11), perineum (9), and extremities (7)	Superficial	Group 1: topical 0.5% timolol maleate solution 40uL/cm2, once daily; Group 2: oral propranolol 1.0 mg/kg, once daily;	6
Li et al., 2014	China	R	43	1–12	NR	Face (43)	Superficial	Group 1: topical 0.5% timolol maleate solution 3 times daily; Group 2: oral propranolol 1.0 mg/kg, once daily;	1–4
Gong et al., 2015	China	R	39	2–9	38.5	Face (39)	Superficial	Group 1: topical 0.5% timolol maleate solution twice daily; Group 2: oral propranolol 1.5 mg/kg, once daily; Group 3: topical 0.5% timolol maleate solution twice daily combined with oral propranolol 1.0 mg/kg, once daily;	6
Chen et al., 2015	China	R	110	1–12	30.9	Face and scalp (78), torso (15), extremities (9), and multiple sites (8)	Superficial, deep, and mixed	Group 1: oral propranolol 1.0 mg/kg, three times daily; Group 2: topical 0.5% timolol maleate solution three times daily combined with oral propranolol 1.0 mg/kg, three times daily;	2
Li et al., 2016	China	R	31	2–11	41.9	Face (31)	Superficial, deep, and mixed	Group 1: oral propranolol 1.5 mg/kg, once daily; Group 2: topical 0.5% timolol maleate solution twice daily combined with oral propranolol 1.0 mg/kg once daily;	8
Zhang et al., 2017	China	R	50	2–18	48	Face (28), scalp (9), torso and extremities (12), and perineum (1)	Superficial	Group 1: topical 0.5% timolol maleate solution, twice daily; Group 2: oral propranolol 1.0–1.5 mg/kg, twice daily;	8
Marey et al., 2018	Egypt	R, SB	25	mean: 5.2	52	Periocular (25)	Superficial	Group 1: oral propranolol 1.0–2.0 mg/kg, once daily; Group 2: topical 0.5% gel twice daily combined with oral propranolol 1.0–2.0 mg/kg once daily;	7
Du et al., 2018	China	R, DB	320	mean: 8.2	38.8	Face and scalp (180), torso (41), extremities (73), and perineum (26)	Superficial, deep, and mixed	Group 1: topical 0.5% timolol maleate solution twice daily; Group 2: oral propranolol 1.0 mg/kg, once daily; Group 3: topical 0.5% timolol maleate solution twice daily combined with oral propranolol 1.0 mg/kg, once daily;	8

RCT, randomized controlled trials; R, randomized; SB, single blinded; DB, double blinded; NR, not reported.

### Study Quality

The details of quality evaluation for the included studies according to the Cochrane assessment tool are listed in **Table 2**. Briefly, one of the included RCTs was a double-blinded study (Du et al., 2018), one was single-blinded (Marey et al., 2018), and the rest were open-label studies (Zhao et al., 2013; Li et al., 2014; Chen et al., 2015; Gong et al., 2015; Li et al., 2016; Zhang et al., 2017). Details of random sequence generation were reported in five studies (Zhao et al., 2013; Gong et al., 2015; Li et al., 2016; Du et al., 2018; Marey et al., 2018). However, none of the studies reported the efforts of allocation concealment. Details of withdrawals and dropouts were reported in all of the studies.

**Table 2:** Quality evaluation of the included RCTs using the Cochrane’s Risk of Bias Tool.

	Random sequence generation	Allocation concealment	Blinding in performance	Blinding in outcome detection	Incomplete outcome data	Reporting bias	Other bias	Total
Zhao et al., 2013	Low	Unclear	Unclear	Unclear	Low	Low	Low	4
Li et al., 2014	Unclear	Unclear	Unclear	Unclear	Low	Low	Low	3
Gong et al., 2015	Low	Unclear	Unclear	Unclear	Low	Low	Low	4
Chen et al., 2015	Unclear	Unclear	Unclear	Unclear	Low	Low	Low	3
Li et al., 2016	Low	Unclear	Unclear	Low	Low	Low	Low	5
Zhang et al., 2017	Unclear	Unclear	Unclear	Low	Low	Low	Low	4
Marey et al., 2018	Low	Unclear	Low	Unclear	Low	Low	Low	5
Du et al., 2018	Low	Unclear	Low	Low	Low	Low	Low	6

### Topical Timolol Versus Oral Propranolol for Treating IH

**Figure 2:**
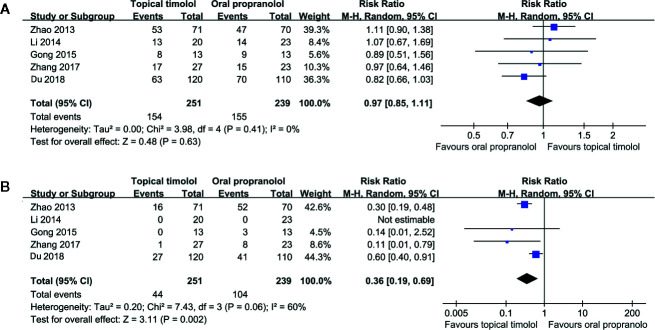
Forest plots for the meta-analyses comparing the efficacy and safety outcomes between topical timolol and oral propranolol for treating IH. **(A)**, response rate after treatment; **(B)**, incidence of adverse events.

Five studies including 490 patients compared the efficacy and safety of topical timolol and oral propranolol for treating IH (Zhao et al., 2013; Li et al., 2014; Gong et al., 2015; Zhang et al., 2017; Du et al., 2018). Pooled results with a random-effect model showed that the response rate of IH patients in the two treatment arms was comparable (RR = 0.97, 95% CI: 0.85 to 1.11; p = 0.63; **Figure 2A**) with no significant heterogeneity (p for Cochrane’s Q test = 0.41, I^2^ = 0%). However, the incidence of adverse events was less in patients allocated to the topical timolol group (RR = 0.36, p = 0.002; **Figure 2B**).

### Combined Therapy Versus Oral Propranolol for Treating IH

**Figure 3:**
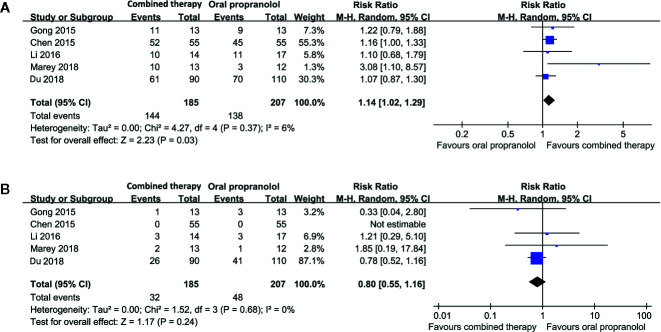
Forest plots for the meta-analyses comparing the efficacy and safety outcomes between the combined therapy and oral propranolol for treating IH. **(A)**, response rate after treatment; **(B)**, incidence of adverse events.

Five studies including 392 patients compared the response rate and adverse events in patients allocated to a combined therapy with topical timolol and oral propranolol and those who received oral propranolol only (Chen et al., 2015; Gong et al., 2015; Li et al., 2016; Du et al., 2018; Marey et al., 2018). Pooled results with a random-effect model showed that combined treatment was associated with a higher response rate compared to oral propranolol alone (RR = 1.14, 95% CI: 1.02 to 1.29; p = 0.03; **Figure 3A**) with no significant heterogeneity (p for Cochrane’s Q test = 0.37, I^2^ = 6%). Moreover, the incidence of adverse events was comparable between the two groups (RR = 0.80, p = 0.24; **Figure 3B**).

### Combined Therapy Versus Topical Timolol for Treating IH

**Figure 4:**
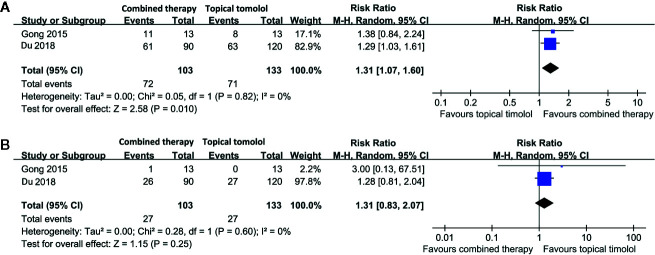
Forest plots for the meta-analyses comparing the efficacy and safety outcomes between the combined therapy and topical timolol for treating IH. **(A)**, response rate after treatment; **(B)**, incidence of adverse events.

Two studies including 236 patients compared the response rate and adverse events in patients allocated to combined therapy with topical timolol and oral propranolol and those who received topical timolol only (Gong et al., 2015; Du et al., 2018). Pooled results with a random-effect model showed that combined treatment was also associated with a higher response rate compared to topical timolol only (RR = 1.31, 95% CI: 1.07 to 1.60; p = 0.01; **Figure 4A**) with no significant heterogeneity (p for Cochrane’s Q test = 0.82, I^2^ = 0%). The incidence of adverse events was comparable between the two groups (RR = 1.31, p = 0.25; **Figure 4B**).

### Publication Bias

**Figure 5:**
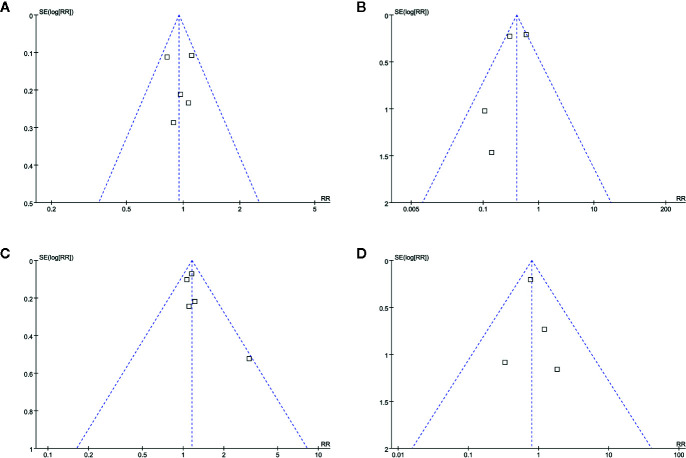
Funnel plots for the meta-analyses. **(A)**, response rate for meta-analysis comparing topical timolol and oral propranolol for treating IH; **(B)**, safety outcome for the meta-analysis comparing topical timolol and oral propranolol for treating IH; **(C)**, response rate for meta-analysis comparing combined therapy and oral propranolol for treating IH; **(D)**, safety outcome for the meta-analysis comparing combined therapy and oral propranolol for treating IH.

The funnel plots for the meta-analyses of safety and efficacy outcomes between topical timolol and oral propranolol, as well as the combined therapy and oral propranolol, were symmetrical on visual inspection (**Figures 5A–D**), indicating low chance of publication biases. Egger’s regression tests were not performed because of the limited number of studies included. The funnel plots for the meta-analyses comparing the efficacy and safety outcomes between the combined therapy and topical timolol were unavailable because only two studies were included. The publication bias for the meta-analyses of each outcome was difficult to estimate because only a maximum of five studies were included.

## Discussion

In this meta-analysis of head-to-head RCTs, we found that topical timolol and oral propranolol showed similar efficacy for treating IH, as indicated by the response rate. However, topical timolol was associated with fewer incidences of adverse events compared to oral propranolol. Interestingly, we also found that combined therapy with topical timolol and oral propranolol was associated with a favorable response rate for treating IH compared to treatment with either topical timolol or oral propranolol alone. Moreover, the combined treatment was not associated with increased risk of adverse events compared to the treatment of topical timolol or oral propranolol alone. Taken together, these findings suggest that combined treatment with topical timolol and oral propranolol may improve treatment efficacy for patients with large or severe IH. For those with mild or moderate IH, topical timolol may be favorable over oral propranolol, considering the better safety profile of timolol.

Although previous meta-analyses have been conducted to evaluate the potential efficacies and safeties of topical timolol and oral propranolol for treating IH, these analyses generally included a limited number of studies (Liu et al., 2015; Zheng and Li, 2018; Yang et al., 2019). More importantly, these studies included both RCTs and non-randomized studies, which may have led to potential bias and therefore could only provide a limited grade of evidence (Zheng and Li, 2018; Yang et al., 2019). In addition, the control groups in these meta-analyses were heterogeneous, including blank treatment, other active medications, and laser therapy, which makes the interpretation of the results difficult (Novoa et al., 2018). In a recent meta-analysis comparing the efficacy between topical timolol and oral propranolol, the authors included two RCTs and concluded that patients who received these two treatment protocols had similar response rates after treatment (Zheng and Li, 2018). Our study confirmed these results by including five RCTs with 490 patients. Moreover, we found that topical timolol was associated with 64% fewer adverse events compared to oral propranolol, indicating better safety profiles of topical timolol. Notably, four RCTs in our meta-analysis comparing the efficacy and safety between topical timolol and oral propranolol included infants with superficial IHs only (Zhao et al., 2013; Li et al., 2014; Gong et al., 2015; Zhang et al., 2017), which implies that for those with mild or moderate IH, topical timolol may be favorable over oral propranolol considering its better safety profile.

To the best of our knowledge our meta-analysis is first study demonstrating that combined treatment with topical timolol and oral propranolol may be more effective than either single treatment protocol in patients with IH without increasing the risk of adverse events. The potential mechanisms underlying the improved efficacy of combined treatments for IHs are unknown. However, in view of the fact that both medications are non-selective β-blockers, the synergetic treatment efficacy may be primarily caused by the combined routes of medicine administration. It has been proposed that multiple hormonal factors, including β-adrenergic catecholamines, epinephrine, norepinephrine, and vascular endothelial growth factors (VEGF) are involved in the pathogenesis and progression of hemangiomas (Boscolo and Bischoff, 2009). β-blockers may confer therapeutic efficacy for IH *via* inhibiting various related signaling pathways and cellular components, including thrombospondin-1, nuclear factor-κB, phosphatidylinositol 3-kinase, protein kinase B, mitogen-activated protein kinase, and hypoxia-inducible factor-1 (Munabi et al., 2016; Lin et al., 2018; Xu et al., 2018). Studies are needed to determine the key mechanisms underlying the therapeutic role of β-blockers for IHs and to explore whether timolol and propranolol have synergetic pharmacological mechanisms that protect against the pathogenesis and progression of hemangiomas. Moreover, it has to be mentioned that although propranolol is currently the first-line treatment for IHs, the efficacy of the drug is around 60% as evidenced in a previous RCT (Leaute-Labreze et al., 2015). Therefore, it could be implied that the blockade of beta1 and beta2 adrenoceptor is probably not sufficient, and that other receptors and other pathways are involved in IH pathogenesis, such as basic fibroblast growth factor, vascular endothelial growth factor and its receptor, glucose transporter isoform 1, matrix metalloproteinase-9, proliferating cell nuclear antigen, type IV collagenase, and angiotensin (Chen et al., 2019). The efficacy of combined treatment with propranolol and drugs targeting these pathways for IHs should also be determined in the future.

Our study has limitations that should be considered when interpreting the results. First, there were limited published RCTs available in the databases and we did not have access to individual patient data. Therefore, we were unable to determine the potential influences of study characteristics on the outcomes, such as the distributions and clinical classifications of the lesions, the dosages, administrative routes, and the treatment durations for each protocol. Future RCTs with adequate sample sizes are warranted to evaluate the potential influence of the above factors on therapeutic efficacies of the combined medications. Secondly, the follow-up durations of the included studies were relatively short. Long-term studies are needed to evaluate the influence of these treatment strategies on the risk of relapse of IH. Thirdly, although the statistical heterogeneity was not significant in most of the outcomes of the meta-analyses (I2 = 0 in 5 of the 6 outcomes), clinical heterogeneity existed among the included studies regarding patient characteristics and treatment protocols et al. Therefore, a random-effect model, the more conservative method, was used to pool the results of these studies in order to generate a more common result. The difference of these aforementioned study characteristics may influence the outcome of the meta-analysis. Finally, some of the included RCTs were open-label and of low quality, which may have introduced potential bias. Our results need to be validated in high-quality RCTs in the future.

In conclusion, combined treatment with topical timolol and oral propranolol may be more effective than either single treatment protocol in patients with IH. However, topical timolol alone conferred similar efficacy for treating IH compared to oral propranolol, but with less incidence of adverse events. A combined treatment with topical timolol and oral propranolol may be considered for patients with large or severe IHs to improve the treatment efficacy. For those with mild or moderate IH, topical timolol may be favorable over oral propranolol.

## Author Contributions

JQ and JjL designed the study. JQ and JjL performed database search, data extraction, and quality evaluation. JQ, DZ, JhL, CC, HY, XL, and BF performed statistical analyses and verified the data. JQ drafted the manuscript. All authors contributed to the article and approved the submitted version.

## Funding

This study was supported by the Medical Science and Technology Research Project of Henan Province (201601015) and the Research Project of Department of Science and Technology of Henan Province (162102310282).

## Conflict of Interest

The authors declare that the research was conducted in the absence of any commercial or financial relationships that could be construed as a potential conflict of interest.
